# Do Digital Diagnostic Probabilities Predict CAMHS Referral Outcomes? A Secondary Analysis of a Multicentre RCT

**DOI:** 10.1192/bjo.2026.11218

**Published:** 2026-06-30

**Authors:** Salah Basheer, Josephine Holland, Kapil Sayal

**Affiliations:** 1 University of Nottingham, Nottingham, United Kingdom; 2 Nottinghamshire NHS Foundation Trust, Nottingham, United Kingdom

## Abstract

**Aims:**

Child and Adolescent Mental Health Services (CAMHS) face unprecedented demand, with many referrals rejected due to incomplete clinical information. Standardized digital tools like the Development and Well-Being Assessment (DAWBA), that generate algorithm-based probable diagnoses from multi-informant symptom reports, could improve referral outcomes. However, thereal-world impact of these tools on referral outcomes remains underexplored. In this context, the current study aimed to examine whether diagnostic probabilities generated by the DAWBA predict referral acceptance in CAMHS. It also explored how the information available to triage team, including referral source, influences referral outcomes.

**Methods:**

We conducted a secondary analysis of anonymised data extracted from a randomized controlled trial conducted across eight NHS Trusts in England. The sample included 483 participants aged 5–17 years with documented DAWBA diagnostic probabilities and referral outcomes. Sociodemographic characteristics and clinical variables were also examined.

**Results:**

Overall, 54.5% (n=263) of referrals were rejected. There were no significant differences in referral acceptance by sex, ethnicity, deprivation, or referral source. Referral acceptance was associated with older age, higher Strength and Deficit Questionnaire (SDQ)parent-rated impact scores, history of previous CAMHS referral, and study recruitment site. Binomial logistic regression showed higher parent impact scores (OR=1.1, 95% CI: 1.01, 1.21) and previous CAMHS referral (OR=2.3, 95% CI: 1.38, 3.69) increased odds of referral acceptance. Referral acceptance also varied with recruitment site. However, a high to very high probability of DAWBA generated diagnoses did not increase chances of referral acceptance with 54% emotional disorders, 78.6% behavioural disorders, or 52.9% comorbid emotional and behavioural disorders being rejected.

**Conclusion:**

This study highlights key gaps in the current CAMHS referral process. While previous CAMHS referral and parent-rated SDQ impact scores were associated with referral acceptance, DAWBA diagnostic probabilities were not. This suggests that children with clinically significant difficulties, identified via standardised assessment, may still face barriers in accessing care. Significant site-level variation in referral acceptance underscores the need for more equitable referral processes.

